# Malondialdehyde–Deoxyguanosine Adducts among Workers of a Thai Industrial Estate and Nearby Residents

**DOI:** 10.1289/ehp.0900907

**Published:** 2009-08-11

**Authors:** Marco Peluso, Petcharin Srivatanakul, Armelle Munnia, Adisorn Jedpiyawongse, Marcello Ceppi, Suleeporn Sangrajrang, Sara Piro, Paolo Boffetta

**Affiliations:** 1 ISPO-Cancer Prevention and Research Institute, Florence, Italy;; 2 National Cancer Institute, Bangkok, Thailand;; 3 National Cancer Institute, Genoa, Italy;; 4 International Agency for Research on Cancer, Lyon, France

**Keywords:** air pollution, lipid peroxidation, M_1_dG adducts, Map Ta Phut, reactive oxygen species

## Abstract

**Background:**

Humans living near industrial point emissions can experience high levels of exposures to air pollutants. Map Ta Phut Industrial Estate in Thailand is the location of the largest steel, oil refinery, and petrochemical factory complexes in Southeast Asia. Air pollution is an important source of oxidative stress and reactive oxygen species, which interact with DNA and lipids, leading to oxidative damage and lipid peroxidation, respectively.

**Objective:**

We measured the levels of malondialdehyde–deoxyguanosine (dG) adducts, a biomarker of oxidative stress and lipid peroxidation, in petrochemical workers, nearby residents, and subjects living in a control district without proximity to industrial sources.

**Design:**

We conducted a cross-sectional study to compare the prevalence of malondialdehyde-dG adducts in groups of subjects experiencing various degrees of air pollution.

**Results:**

The multivariate regression analysis shows that the adduct levels were associated with occupational and environmental exposures to air pollution. The highest adduct level was observed in the steel factory workers. In addition, the formation of DNA damage tended to be associated with tobacco smoking, but without reaching statistical significance. A nonsignificant increase in DNA adducts was observed after 4–6 years of employment among the petrochemical complexes.

**Conclusions:**

Air pollution emitted from the Map Ta Phut Industrial Estate complexes was associated with increased adduct levels in petrochemical workers and nearby residents. Considering the mutagenic potential of DNA lesions in the carcinogenic process, we recommend measures aimed at reducing the levels of air pollution.

Humans living in urban areas situated near industrial point emissions, such as steel factories and petrochemical complexes, can experience high levels of exposure to air pollutants (Kibble and Harrison 2005; Peluso et al. 2008), including a variety of known carcinogens (Cohen 2000). Map Ta Phut Industrial Estate (MIE) in Rayong Province, Thailand, is the location of the largest steel, oil refinery, and petrochemical factory complexes in Southeast Asia. MIE complexes produce mixtures of air pollutants containing nitrogen dioxide, ozone, propylene, ethylene, benzene, and polycyclic aromatic hydrocarbons (PAH) together with particulate matter onto which compounds are absorbed (Bakker et al. 2000; Bergamaschi et al. 2005; Boogard and Van Sitter 1994; Kim et al. 2005; Sørensen et al. 2003; Tang et al. 2006; Yang et al. 2002).

We have recently undertaken a cross-sectional study to evaluate the formation of bulky DNA adducts, a biomarker of PAH exposure (Peluso et al. 2001), in people living and working in Map Ta Phut (MTP) (Peluso et al. 2008). That study showed an increased formation of bulky DNA adducts in the MIE workers with respect to those living in Rayong Province. Furthermore, the subjects living near the MIE complexes experienced an excess of bulky DNA adduct formation relative to residents of a control district in the same province.

Air pollution is also an important source of oxidative stress and reactive oxygen species (ROS), which can interact with DNA and lipids, leading to oxidative damage and lipid peroxidation (LPO), respectively (Buthbumrung et al. 2008; Calderón-Garcidueñas et al. 1999; Chen et al. 2007; Møller et al. 2008; Singh et al. 2007; Sørensen et al. 2003).

Malondialdehyde (MDA) is a natural product of LPO, which is also formed during prostaglandin E_2_ biosynthesis via cyclooxygenase (Marnett 1999). MDA is an aldehyde capable of interacting with DNA to form exocyclic adducts, including 3-(2-deoxy-β-d-erythro-pentafuranosyl)pyrimido[1,2-α]purin-10(3H)-one deoxyguanosine (M_1_dG). This adduct can be also generated through base propenal intermediates (Jeong and Swenberg 2005). The importance of M_1_dG adducts in carcinogenesis is emphasized by their ability to induce base pair mutations and cause frameshift mutations in reiterated sequences (VanderVeen et al. 2003).

A physiologic background of M_1_dG adducts has been reported in a number of human tissues, including breast, colon, and bronchial mucosa, which seems to be influenced by individual susceptibility and environmental factors, including dietary and lifestyle habits (Fang et al. 1996; Leuratti et al. 2002; Munnia et al. 2006; Wang et al. 1996). For instance, we and others found that the formation of M_1_dG adducts is associated with smoking habit in laryngeal, bronchial, and oral mucosa (Munnia et al. 2004, 2006; Zhang et al. 2002). Furthermore, population-based studies suggest that increased levels of M_1_dG adducts can be related to cancer development and tumor progression (Munnia et al. 2004, 2006; Wang et al. 1996).

In the present study, we evaluated whether air pollution emitted from MIE complexes was associated with levels of M_1_dG adducts, a biomarker of oxidative stress and LPO. Our approach consisted of a cross-sectional study to compare the prevalence of DNA damage in groups of subjects experiencing various degrees of air pollution exposures (Peluso et al. 2008). This was done by measuring the amount of M_1_dG adducts in MIE workers, nearby residents, and subjects living in a control district in the same province (Rayong) but without proximity to industrial sources. The levels of M_1_dG adducts were measured using the ^32^P-DNA postlabeling technique (Munnia et al. 2006).

## Materials and Methods

### Study subjects

Study subjects working in MIE complexes were identified and recruited by the industrial health service. MTP participants who were nearby residents or living in a control district from the Rayong Province without proximity to industrial sources were contacted and recruited by local health personnel. Only control subjects without occupational history in industries entailing exposure to known or suspected carcinogens were eligible in the MTP study. The study population comprised three groups of subjects: *a*) MIE workers, *b*) nearby residents, and *c*) residents in a control district. Ninety-five percent of the eligible residents in each study group participated in the MTP study. Written consent to participate in the MTP study was given by all subjects fulfilling inclusion criteria after they were given a detailed description of sampling procedures and the aims of the project. The MTP study was approved by the relevant ethical committee. A questionnaire concerning occupational history, smoking habit, and diet was administered to study subjects before blood sampling.

### Preparation of the reference M_1_dG adduct standards

We prepared two reference adduct standards using MDA or hydrogen peroxide (H_2_O_2_). For MDA, calf thymus (CT) DNA or leukocyte DNA from a blood donor was treated with 10 mM MDA as described by Sun et al. (2004), yielding M_1_dG (Leuratti et al. 1998; Sun et al. 2004; Vaca et al. 1995). For H_2_O_2_, the epithelial lung carcinoma cell line A549 was exposed to 100 μM H_2_O_2_. MDA-treated CT DNA was then diluted with untreated CT DNA to obtain decreasing levels of the reference adduct standard to generate a calibration curve (*R*^2^ = 0.99).

### Measurement of M_1_dG adducts in peripheral leukocytes

We measured the levels of M_1_dG adducts using our previously described method (Munnia et al. 2006) with minor modifications. DNA (1–2 μg) was digested by micrococcal nuclease and spleen phosphodiesterase. Hydrolyzed samples were treated with nuclease P1 (2.5 μg) for 30 min at 37°C. The nuclease P1-treated samples were incubated with 15–25 μCi [γ-^32^P]- ATP and T4-polynucleotide kinase (0.75 U/μL) to generate labeled M_1_dG adducts. Samples were applied to the origin of chromatograms and developed with 0.35 MgCl_2_ up to 2.0 cm on a filter paper wick. Plates were developed in the opposite direction with 2.1 M lithium formate, 3.75 M urea (pH 3.75), and then run at the right angle to the previous development with 0.24 M sodium phosphate, 2.4 M urea (pH 6.4).

We detected and quantified M_1_dG adducts and total nucleotides using the storage phosphor imaging technique, which employs intensifying screens (Molecular Dynamics, Sunnyvale, CA, USA) for 0.20–48 hr. The screens were scanned using a Typhoon 9210 (Amersham, Little Chalfont, Buckinghamshire, UK). To process the data, we used ImageQuant (Molecular Dynamics, Sunnyvale, CA, USA). After background subtraction, the levels of M_1_dG adducts were expressed as relative adduct labeling [screen pixels in adducted nucleotides ÷ screen pixels in total normal nucleotides (NN)]. To calculate the levels of total NN, aliquots of hydrolyzed DNA were appropriately diluted and reacted in the mixtures used for M_1_dG adduct labeling. The ^32^P-labeled total nucleotides obtained were separated on Merck PEI-cellulose TLC plates using 280 mM ammonium sulfate, 50 mM sodium phosphate. The values measured for the M_1_dG adducts were corrected across experiments based on the recovery of the internal standard after the ^32^P-DNA postlabeling assay.

### Statistical analysis

All statistical analyses were performed on log-transformed data to stabilize the variance and normalize the distribution of M_1_dG adducts. MIE workers and nearby residents were grouped according to tertiles for duration of employment and years spent at residence before statistical analyses.

We initially performed a descriptive analysis to explore the relationship between individual variables and M_1_dG adducts. In the univariate setting, the mean levels of DNA adducts across the levels of each variable, that is, age, sex, smoking habit, residence, employment, duration of employment (among petrochemical workers), and years of residence (among nearby residents), were compared by analysis of variance. Post hoc Dunnett tests were performed for multiple comparisons among such variable levels.

The multivariate analysis was then performed using log-normal regression models including terms for type of exposure, age, sex, smoking habit, years spent at the residence, and duration of employment to estimate the effect of each variable on the outcome, adjusting for the concomitant effect of the other variables included in the model. The regression parameters estimated from the model are interpreted as ratios between the means of M_1_dG adducts of each level of the study variables with respect to the reference level, adjusted by age, sex, and smoking habit. A *p*-value of < 0.05 (two-tailed) was considered significant for all of the tests. The data were analyzed using SPSS 13.0 (SPSS, Chicago, IL, USA).

## Results

### Reference M_1_dG adduct standards

We first evaluated whether MDA treatment was capable of inducing the formation of M_1_dG adducts in CT DNA *in vitro*. A statistically significant increased formation of M_1_dG adducts was found in MDA-treated DNA relative to control DNA (*p* < 0.001). The mean levels of M_1_dG adducts per 10^6^ NN ± SE were 0.32 ± 0.06, 1.6 ± 0.23, and 5.0 ± 0.45 in 1 mM, 4 mM, and 10 mM MDA-treated DNA, respectively, while a mean of 0.06 M_1_dG adducts per 10^6^ NN (± 0.01) was detected in untreated DNA.

**Figure 1:**
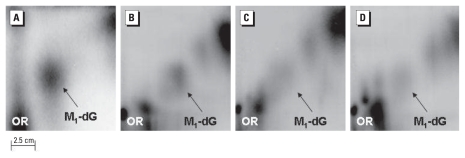
Chromatograms showing the pattern of M_1_dG adducts in 10 mM MDA-treated human leukocyte DNA (*A*); and the pattern of M_1_dG adducts in the leukocytes of MIE workers (*B*), nearby residents (*C*), and subjects living in a control district of Rayong Province without industrial exposures (*D*). OR, origin. The bar shown in (*A*) applies to all four chromatograms.

In a subsequent experiment, we analyzed whether MDA treatment was capable of causing adduct formation in human leukocyte DNA. We found a statistically significant increase in M_1_dG adducts (Figure 1A) relative to control DNA (*p* < 0.001). The mean level of M_1_dG adducts per 10^6^ NN ± SE was 2.2 ± 0.6 and 0.02 ± 0.01 in MDA-treated and untreated DNA, respectively. This adduct spot was previously identified as M_1_dG using different techniques (Leuratti et al. 1998; Sun et al. 2004; Vaca et al. 1995). The presence of a background adduct spot in the untreated samples is in keeping with previous results reporting background levels of M_1_dG adducts in control DNA (Leuratti et al. 1998; Sun et al. 2004).

Next, we analyzed whether free radicals were capable of inducing the same DNA lesion in an *in vitro* system by incubating epithelial lung carcinoma cell line A549 with H_2_O_2_. Our findings showed that treatment with 100 μM H_2_O_2_ induced a statistically significant increase in M_1_dG adducts in the lung carcinoma cells relative to the unexposed cells (*p* < 0.001). The average level of M_1_dG adducts per 10^6^ NN was 0.25 ± 0.09 and 0.07 ± 0.01 in H_2_O_2_-treated and untreated cells, respectively.

### M_1_dG adducts in the leukocytes of MTP study subjects

We measured the formation of M_1_dG adducts in the leukocyte DNA of 173 subjects: 38.73% were MIE workers, 33.53% were nearby residents, and 27.74% were living in a control district of the Rayong Province. The mean age of MIE workers, MIE residents, and district controls was 31.6 ± 6.7 years, 36.2 ± 8.5 years, and 34.3 ± 6.8 years, respectively. There was a higher proportion of males than females (80.3%); 38.2% of participants were classified as nonsmokers, 4.0% as former smokers, and 57.8% as current smokers, based on smoking during the 3 months before blood sampling.

Figure 1 shows the pattern of M_1_dG adduct spots in the chromatograms of study participants. The intensity of the adduct spot was stronger in the plates of MIE workers and nearby residents compared with chromatograms of subjects living in the control district.

Table 1 reports the distributions of demographic characteristics and mean levels of M_1_dG adducts according to exposure group. Adduct levels were higher in MIE workers and nearby residents than in the control group. The mean levels of M_1_dG adducts of current smokers were increased, but without reaching statistical significance, in nearby residents and in the control group. No effect of smoking was observed in MIE workers.

Table 2 shows the mean levels of M_1_dG adducts per 10^8^ NN ± SE in the MTP study according to residence and employment, years of employment (among petrochemical workers), and years of residence (among nearby residents) and for sex, age, and smoking habit for the whole population plus adjusted ratios of mean adduct levels based on the multivariate regression model.

Observed (unadjusted) mean levels of M_1_dG adducts per 10^8^ NN ± SE of MIE workers (6.0 ± 0.5) and nearby residents (3.7 ± 0.4) were significantly higher than those of subjects living in the control district (2.9 ± 0.4; *p*-values < 0.001 and 0.014, respectively). Steel factory workers had the highest levels of M_1_dG adducts (6.4 ± 0.7;*p* < 0.001). When recent smoking habit (within 3 months) was considered, the adduct levels of current smokers (4.8 ± 0.4) were significantly higher that those of nonsmokers (3.7 ± 0.4, *p* = 0.027). A nonsignificant increase in adduct levels was associated with duration of employment. No other univariate effects were observed.

The results of the multivariate analysis confirm that the levels of M_1_dG adducts were significantly higher among MIE workers with respect to the control groups. Moreover, adduct levels of nearby residents were significantly higher than those of subjects living in the control district. The highest adduct level was observed in the steel factory workers. The formation of DNA damage tended to be associated with tobacco smoking, but without reaching statistical significance. A nonsignificant increase in M_1_dG adducts was associated with 4–6 years of employment, but adduct levels were not associated with longer duration of employment. The adduct formation of MIE workers seems to reach some kind of saturation point for longer duration. No association with the duration of nearby residence was found.

## Discussion

One of the largest steel, oil refinery, and petrochemical factory complexes in Southeast Asia has been located in MTP (Thailand) since 1988. Coal power plants and oil power plants, capable of using several by-products including petroleum coke derived from oil refinery coker units or other cracking processes, are located at the MIE site for power generation. Complex mixtures of air pollutants, including benzene, toluene, benzo(*a*)anthracene, benzo(*a*)chrysene, and transition metals are produced from such petrochemical installations and energy plants (Bakker et al. 2000; Bergamaschi et al. 2005; Roma-Torres et al. 2006). Steel factories are able to melt tons of metal per year, generating high PAH emissions (Yang et al. 2002).

In the present study, average levels of M_1_dG adducts were significantly higher in MIE workers than in control groups. In addition, adduct levels were highest among the individuals working in factories with industrial processes characterized by emissions of PAH and transition metals, such as steel factories. Most likely, steel workers have increased exposure to iron, which can promote Fenton chemistry leading to oxidative stress. Finally, the mean level of M_1_dG adducts among nearby residents was significantly higher than the mean level among subjects living in a control district without proximity to industrial sites.

The industrial emissions from MIE complexes are the most important source of air pollutants in the MTP area and are therefore likely to be involved in the increased adduct levels observed among MTP residents. Indeed, industrial air pollution constituents can induce DNA damage in a number of ways, including through production of ROS, which can initiate LPO and cause an intracellular excess of MDA. ROS can also induce the production of M_1_dG adducts through deoxyribose oxidation. For instance, benzene-derived quinones are reactive intermediates with the ability to redox-cycle, which is a reaction that generates superoxide, H_2_O_2_, and hydroxyl radicals (Bolton et al. 2000; Sørensen et al. 2003).An alternative mechanism by which air pollutants can induce DNA damage involves the action of transition metals, such as iron, copper, and chromium, on the surface of the particulate matter, which produces ROS through the Fenton reaction (Sørensen et al. 2003). Finally, air pollution exposure can activate macrophages and neutrophils that release ROS, such as H_2_O_2_ and hypochlorite acid.

Several studies have showed higher levels of oxidative DNA damage among subjects exposed to air pollution (Sørensen et al. 2003). Increased LPO, such as measured by MDA or 8-iso-prostaglandin-F_2α_, was detected in individuals exposed to air pollutants (Chen et al. 2007; Sørensen et al. 2003; Yang et al. 2007). High levels of M_1_dG adducts were also reported in urban workers from Sofia, Bulgaria, but not in the police officers from Kosice, Slovac Republic, and Prague, Czech Republic (Singh et al. 2007). Admittedly, we realize that our study could ideally have been conducted at the level of bronchi, which are more exposed to airborne carcinogens and more competent in terms of metabolic activation. However, although leukocytes are not the direct target of environmental carcinogens, the level of carcinogen–DNA adducts in leukocytes correlates with carcinogen-induced damage in human lung tissues (Peluso et al. 2005; Tang et al. 2001; Wiencke et al. 1995). In addition, cells from peripheral blood that migrate and circulate through the lung can be exposed to accumulated unmetabolized toxic compounds in this tissue (Wiencke 2002).

In the present study, the formation of M_1_dG seems to saturate among the petrochemical workers that have been exposed for several years to the air pollutants emitted from the petrochemical complexes. Indeed, the levels of M_1_dG adducts tended to increase after 4–6 years of employment and to reach a steady-state level after 7 years. Conversely, we did not observe any effects with duration of nearby residence. Higher exposure levels may be necessary to induce similar effects in environmentally exposed residents.

Table 3 compares the levels of M_1_dG adducts found in the present study with those detected by different laboratory methods in DNA of leukocytes as reported in the literature. The levels of M_1_dG adducts detected in the present study are in keeping with those determined in the leukocytes of human volunteers by immunoaffinity purification gas chromatography/electrochemical detection negative chemical ionization/mass spectrometry (imafin/GC-MS NCI/MS), immuno-enriched ^32^P-postlabeling, and immunoslot blot techniques (Leuratti et al. 1998; Rouzer et al. 1997; Sun et al. 2004). Higher M_1_dG adduct levels were reported in a multicenter occupational study in Eastern Europe and in Finnish volunteers by an earlier ^32^P-postlabeling/reverse-phase high-performance liquid chromatography (HPLC) technique and an immunoslot blot method (Singh et al. 2007; Vaca et al. 1995).

Current smokers inhale a broad range of carcinogens and ROS derived from tobacco pyrolysis products, which can lead to M_1_dG adduct formation in a number of ways. In addition, the relatively long half-life of M_1_dG adducts of 12.5 days (Marnett 1999) renders it a potentially interesting biomarker of oxidative stress and carcinogen exposures, including recent smoking. Thus, we analyzed the association between levels of M_1_dG adducts and smoking. We found a significant difference between the levels of DNA damage of smokers and nonsmokers using univariate analysis. However, the effect was less evident in the multivariate analysis. The association between smoking habit and M_1_dG adduct levels may have been confounded by air pollution or other factors. This may explain why an effect of tobacco smoking on adduct levels was no longer evident in results from the adjusted regression model.

We previously examined the relationship between smoking and endogenous DNA adducts and found higher values of bronchial and laryngeal DNA damage in smokers (Munnia et al. 2004, 2006). The effects of smoking on bronchial DNA adducts also persisted when urinary thiocyanate was used to measure the extent of exposure to tobacco smoke (Munnia et al. 2006). Similar relationships have been reported in the oral mucosa of tobacco smokers (Zhang et al. 2002), but other investigators reported no differences for smoking habit in breast and colon mucosa (Leuratti et al. 2002; Wang et al. 1996).

The potential effect of MIE emissions on the health of nearby residents has been widely studied in recent years. An increased cancer incidence was reported in the MTP area (Peluso et al. 2008). Age-standardized incidence rates for all cancers in 1997–2001 were 181.0 in men and 183.9 in women in the MTP area compared with 122.6 and 116.8 in the rest of the Rayong Province. An excess risk of respiratory diseases was also found in nearby residents (Jadsri et al. 2006). We recently showed that MIE workers and nearby residents can experience an excess formation of bulky DNA adducts, a biomarker of PAH exposure possibly related to lung cancer risk (Peluso et al. 2005). In the present study, we found that levels of M_1_dG adducts (a biomarker of oxidative stress and LPO) also were increased in MIE workers and nearby residents.

Oxidative stress-induced DNA damage is an important marker of air pollution exposure (Sørensen et al. 2003). Furthermore, oxidative stress and LPO are thought to underlie the etiology of many cancers (Leuratti et al. 2002; Munnia et al. 2004, 2006; Wang et al. 1996). For instance, we observed that higher levels of endogenous DNA adducts were increased in lung cancer cases compared with controls, but only in smokers (Munnia et al. 2006). In addition, lung cancer cases with levels of MDA–DNA adducts above the population median had reduced survival, although not statistically significant, after adjusting for age, sex, and smoking habit (Munnia et al. 2006).

## Conclusions

Air pollution exposure was associated with increased formation of M_1_dG adducts, a biomarker of oxidative stress, and LPO in MIE workers and nearby residents compared with individuals living in a nonindustrial control district. Thus, considering the mutagenic potential of the DNA lesions in the carcinogenic process, we recommend measures of air pollution control aimed at reducing the levels of air pollutants in the MTP area.

## Figures and Tables

**Table 1:** Distributions and percentage of demographic characteristics and the levels of M_1_dG adducts (mean ± SE) by exposure group.

	Control district residents		Nearby residents		Petrochemical workers	
	No. (%)^a^	Mean ± SE^b^	No. (%)^a^	Mean ± SE^b^	No. (%)^a^	Mean ± SE^b^
Sex						
Male	37 (77.1)	3.1 ± 0.5	44 (75.9)	3.7 ± 0.6	58 (86.6)	6.0 ± 0.5
Female	11 (22.9)	2.3 ± 0.7	14 (24.1)	3.8 ± 0.7	9 (13.4)	5.7 ± 1.7
Age (years)						
≤ 31	18 (37.5)	3.1 ± 0.6	17 (29.3)	3.2 ± 0.7	35 (52.2)	6.1 ± 0.7
32–38	13 (27.1)	1.7 ± 0.4	25 (43.1)	3.3 ± 0.4	24 (35.8)	5.5 ± 0.5
≥ 39	17 (35.4)	3.5 ± 0.9	16 (27.6)	5.1 ± 1.5	8 (11.9)	7.1 ± 2.5
Smoking						
Nonsmokers	26 (54.2)	2.1 ± 0.4	23 (39.7)	3.3 ± 0.5	17 (25.4)	6.7 ± 1.2
Former smokers	–	–	3 (5.2)	3.6 ± 1.1	4 (6.0)	4.7 ± 1.1
Smokers	22 (45.8)	3.8 ± 0.7	32 (55.2)	4.1 ± 0.8	46 (68.7)	5.9 ± 0.6

a Some figures do not add up to the total because of missing values.

b Levels of adducts per 10^8^ NN.

**Table 2:** M_1_dG adducts (mean ± SE) and the parameter estimates of the multivariate regression model.

Study variables	No. (%)^a^	Mean ± SE^b^	Means ratio^c^	95% CI	*p*-Value
Sex					
Male^d^	139 (80.3)	4.5 ± 0.3	1.00	–	–
Female	34 (19.7)	3.8 ± 0.6	0.96	0.61–1.52	0.861

Age (per year)	173 (100)	4.4 ± 0.3	1.01	0.99–1.03	0.444

Smoking					
Nonsmokers^d^	66 (38.2)	3.7 ± 0.4	1.00	–	–
Former smokers	7 (4.0)	4.2 ± 0.7	1.09	0.65–1.84	0.732
Current smokers	100 (57.8)	4.8 ± 0.4	1.23	0.86–1.76	0.247

Residence and employment					
Control district residents^d^	48 (27.7)	2.9 ± 0.4	1.00	–	–
Nearby residents	58 (33.5)	3.7 ± 0.4	1.65	1.06–2.56	0.027
All workers	67 (38.8)	6.0 ± 0.5	3.03	2.00–4.60	< 0.001
Refinery workers	21 (12.1)	5.3 ± 0.8	2.63	1.57–4.40	< 0.001
Tinplate workers	13 (7.5)	6.0 ± 1.2	2.93	1.67–5.14	< 0.001
Steel factory workers	33 (19.2)	6.4 ± 0.7	3.35	2.17–5.18	< 0.001

Employment in the industrial complexes (years)					
≤ 3^d^	16 (29.6)	4.9 ± 0.9	1.00	–	–
4–6	18 (33.3)	6.3 ± 0.9	1.22	0.78–1.90	0.382
≥ 7	20 (37.1)	6.3 ± 1.1	1.08	0.63–1.86	0.768

Residence near the industrial complexes (years)					
≤ 5^d^	22 (37.9)	3.3 ± 0.5	1.00	–	–
6–15	17 (29.3)	3.6 ± 1.0	0.77	0.38–1.55	0.452
≥ 16	19 (32.8)	4.4 ± 1.0	1.00	0.59–1.69	0.998

CI, confidence interval. For the parameter estimates, the effect of each variable (means ratio) is the ratio between the mean adducts of each level of study variables with respect to the reference level, adjusted by age, sex, and smoking habit.

a Some figures do not add up to the total because of missing values.

b Levels of adducts per 10^8^ NN.

c Separate models were used to estimate associations according to residence and type of employment, duration of employment (among industrial workers only; *n* = 54), and duration of residence near the industrial complex (among nearby residents only; *n* = 58), with adjustment for sex, age, and smoking.

d Reference level.

**Table 3:** Levels of M_1_dG adducts detected by different methods in DNA of leukocytes, as reported in the literature, and compared with those detected in the MTP study.

Detection method	DNA (μg)	Population	Country	No.	Mean^a^	References
^32^P-postlabeling/reverse-phase HPLC	10	Human volunteers	Finland	26	26	Vaca et al. 1995

Imafin/GC-MS NCI/MS	1,000	Human volunteers	USA	10	6.2	Rouzer et al. 1997

Immunoslot blot	1.0	Human volunteers	Great Britain	8	5.6–9.5	Leuratti et al. 1998

Immuno-enriched ^32^P-postlabeling	5–10	Human volunteers	Germany	26	9.5	Sun et al. 2004

Immunoslot blot	1.0	Unexposed controls	Czech Republic	51	37.1	Singh et al. 2007
		City policemen	Czech Republic	52	32.4	
		Unexposed controls	Slovakia	55	20.0	
		City policemen	Slovakia	51	17.8	
		Unexposed controls	Bulgaria	50	31.2	
		Policemen and bus drivers	Bulgaria	95	41.5	

^32^P-Postlabeling	1–2	Control district residents	Thailand	48	2.9	Present study
		Nearby residents	Thailand	58	3.7	
		Occupationally exposed	Thailand	67	6.0	

a Level of adducts per 10^8^ NN.
